# Supplementary material for: Epidemiology and Molecular Transmission Characteristics of HIV in the Capital City of Anhui Province in China
Source: Pathogens. 2021 Nov 29;10(12):1554. doi: 10.3390/pathogens10121554 (PMC8708547; doi:10.3390/pathogens10121554)
Supplement: Supplementary file 1 [file pathogens-10-01554-s001.zip › pathogens-1461114-supplementary.pdf]

Supplementary Table S1. The sequence distribution of each province of the dataset B

Region	N	Sampling year	Province*
Northeast	1714	2000-2019	HLJ (459), LN (787), JL (468)
North	11300	2003-2019	BJ (8623), HE (1818), NM (210) , SX (512), TJ (137)
East	9914	2004-2020	AH (2714), FJ (223), HF (816), JS (1686), JX (419), SH (1851), SD (981), ZJ (1224)
South	9844	2005-2019	HI (198), GD (6519), GX (3127)
Center	4944	2001-2019	HA (2463), HB (1011), HN (1470)
Northwest	2453	2003-2019	GS (238), NX (105), QH (69), SN (734), XJ (1307)
Southwest	10629	2000-2019	CQ (1060), GZ (860), SC (4437), YN (4172), XZ (100)
Overall	50798	2000-2020	

*Province: Anhui (AH), Beijing (BJ), Chongqing (CQ), Fujian (FJ), Guangdong (GD), Gansu (GS), Guangxi Zhuang Autonomous Region (GX), Guizhou (GZ), Henan (HA), Hubei (HB), Hebei (HE), Hefei (HF), Hainan (HI), Heilongjiang (HLJ), Hunan (HN), Jilin (JL), Jiangsu (JS), Jiangxi (JX), Liaoning (LN), Inner Mongolia Autonomous Region (NM), Ningxia Hui Autonomous Region (NX), Qinghai (QH), Sichuan (SC), Shandong (SD), Shanghai (SH), Shaanxi (SN), Shanxi (SX), Tianjin (TJ), Xinjiang Uygur Autonomous Region (XJ), Tibet Autonomous Region (XZ), Yunnan (YN), Zhejiang (ZJ).
